# Hepatotoxicity and hematologic complications induced by fusidic acid in a patient with hepatitis B cirrhosis: A case report

**DOI:** 10.1097/MD.0000000000017852

**Published:** 2019-11-11

**Authors:** Zhong-Fang He, Lin Chen, Jian-Ping Zhang, Qing-qing Wang

**Affiliations:** a Department of Pharmacy; b Department of Infectious Disease, First Hospital of Lanzhou University, Lanzhou; c Department of Pharmacy, Yanan University Affiliated Hospital, Yanan, China.

**Keywords:** adverse effect, fusidic acid, jaundice, neutropenia, thrombocytopenia

## Abstract

**Rationale:**

Fusidic acid (FA) is an active agent against gram-positive bacteria such as *Staphylococcus*, it is generally well tolerated and the major adverse effects are mild gastrointestinal discomfort, diarrhea, and headache. However, some rare side effects such as granulocytopenia and thrombocytopenia have also been reported. Here we report a case of FA-induced hepatotoxicity and hematologic toxicity.

**Patient concerns:**

A 54-year-old woman with hepatitis B cirrhosis was referred to us because of fever, *Staphylococcus aureus* was identified in the twice blood culture, and intravenous FA was given (0.5 g, q8 hours). Twelve days after FA therapy, she developed nausea and jaundice. Meanwhile, complete blood cell count showed neutropenia (white blood cell count of 1360/μL, neutrophil of 619/μL) and aggravated thrombocytopenia (platelet count of 18,000/μL). Adverse drug reaction was suspected, and FA was stopped immediately, after 1 day of discontinuation of FA, nose bleeding occurred and the platelet count declined further and reached the lowest value of 4000/μL.

**Diagnoses:**

Hepatotoxicity and hematologic complications induced by FA were diagnosed.

**Interventions and outcomes:**

The FA was stopped immediately, and concentrated platelet transfusion was used. Five days after withdrawal of FA, jaundice resolved and the hematologic index returned to the level before the medication.

**Lessons:**

Hematologic adverse effect accompanying with hepatotoxicity may be induced by FA. Though the risk is rather low, it should not be overlooked.

## 1 Introduction

Fusidic acid (FA) is an active agent against gram-positive bacteria such as *Staphylococcus*, and it is used in hospital as the 2nd-line therapy for methicillin-resistant *Staphylococcus aureus* infection. FA is generally well tolerated and the major adverse effects are mild gastrointestinal discomfort, diarrhea, and headache. Hepatotoxicity and jaundice induced by FA have been reported. The hematologic side effects such as granulocytopenia and thrombocytopenia have also been rarely reported.^[1]^ Here we describe a case of FA-induced jaundice, neutropenia, and aggravated thrombocytopenia in a patient with hepatitis B cirrhosis.

## 2 Case report

**Figure 1:**
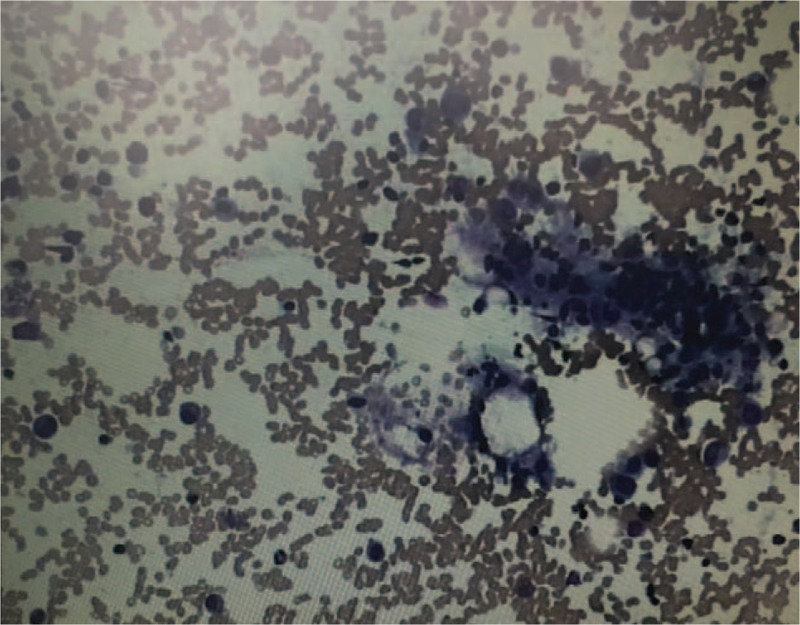
Bone marrow biopsy showed myeloid and megakaryocyte hyperplasia after 12 days of fusidic acid treatment.

**Figure 2:**
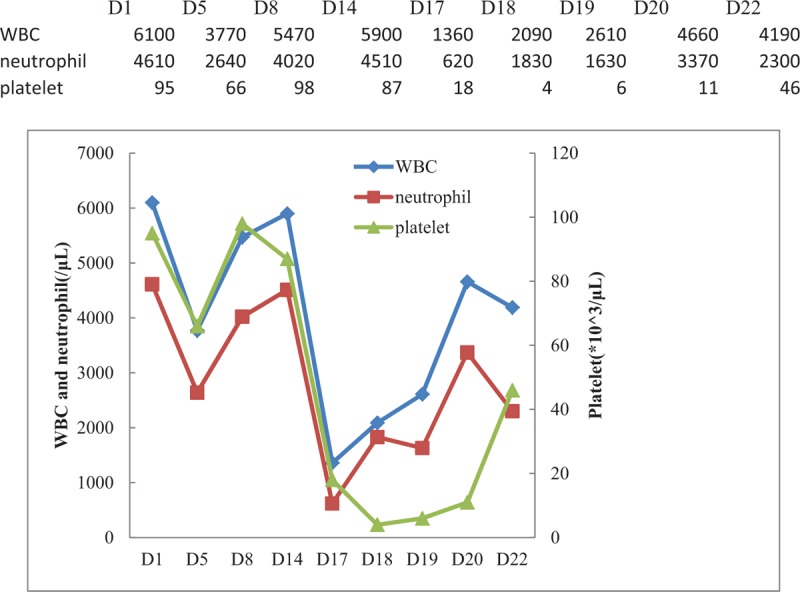
Changes in white blood cell (WBC), neutrophil, and platelet counts during hospitalization. D = day, D1 = the date of admission, D5 = the date of initiation of fusidic acid (FA) therapy, D17 = the date of cessation of FA therapy, the normal reference range of WBC 4000 to 10,000/μL, neutrophil 2000 to 7000/μL, and platelet 100 to 300 × 10^3^/μL.

**Figure 3:**
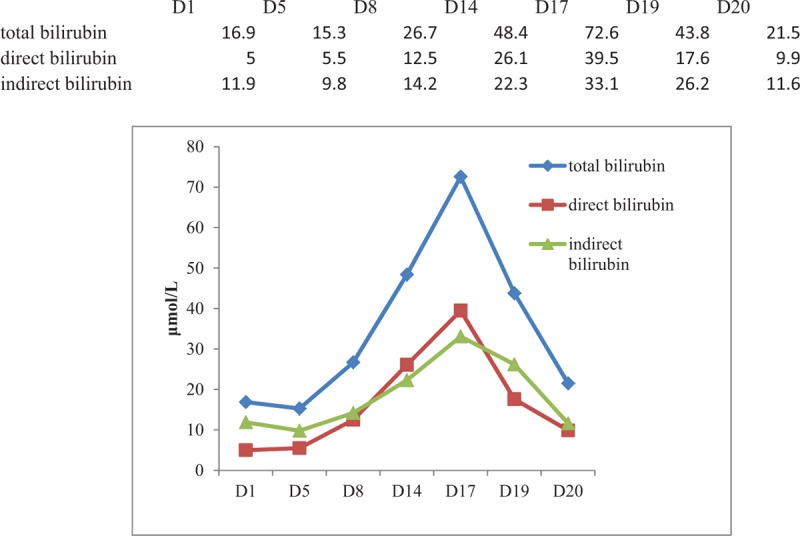
Changes of total bilirubin, direct bilirubin, and indirect bilirubin during hospitalization. D = day, D1 = the date of admission, D5 = the date of initiation of fusidic acid (FA) therapy, D17 = the date of cessation of FA therapy, the normal reference range of total bilirubin 9.1 to 30.1 μmol/L, direct bilirubin 0 to 6.8 μmol/L, indirect bilirubin 0 to 19 μmol/L.

A 54-year-old woman with hepatitis B cirrhosis, weighing 60 kg, presented to the hospital because of fever for half a month. On examination, she was conscious, the temperature was 36.6°C (the maximum body temperature was 39.0°C during the course of fever), pulse 74 times/min, R 18 times/min, and blood pressure 125/70 mm Hg. She had no history of drug allergies or adverse drug reactions. The temperature of patient did not improve after 3 days of empirical anti-infective therapy with piperacillin/tazobactam (PT), at this time, *S aureus* was identified in the twice blood culture. Additional laboratory analyses revealed the following: white blood cell (WBC) count of 3770/μL, neutrophil of 2639/μL, platelet of 66,000/μL, and hemoglobin of 10.8 g/dL, serum total bilirubin was 15.3 μmol/L, direct bilirubin 5.5 μmol/L, indirect bilirubin 9.8 μmol/L, alanine aminotransferase (ALT), aspartate aminotransferase (AST), and kidney function were normal. Under the impression of bacteremia, PT was replaced by intravenous FA (0.5 g, q8 hours). Twelve days after FA therapy, she developed nausea and jaundice. And liver function test showed serum total bilirubin was 72.6 μmol/L, direct bilirubin 39.5 μmol/L, indirect bilirubin 33.1 μmol/L, ALT, AST, and kidney function were still normal. Complete blood cell count showed aggravated thrombocytopenia with platelet count of 18,000/μL, neutropenia with neutrophil count of 619/μL, and WBC of 1360/μL, and hemoglobin of 12.8 g/dL. On suspicion of adverse drug reaction, FA was stopped immediately, reduced glutathione was prescribed for hepatoprotective therapy. Given that the patient's temperature remained normal for 10 days, other antibiotic was not used. One day after discontinuation of FA, nose bleeding occurred with a nadir of platelet count of 4000/μL. However, WBC count increased slightly to 2090/μL, neutrophil to 1831/μL. Due to the severity of low platelet count, transfusion of random-donor platelet concentrates was used, but thrombocytopenia did not improve immediately. At the same time, bone marrow aspiration showed myeloid and megakaryocytic hyperplasia, suggesting increasing peripheral destruction (Fig. 1). Five days after cessation of FA, jaundice resolved and the hematologic index returned to the level before FA. Throughout the period of FA treatment and after discontinuation of FA, the other drugs she took concurrently were entecavir tablets and polyene phosphatidylcholine injection, the former had been taking for 2 months prior to this admission, the latter was administered for 22 days from this admission to discharge. The serial data of WBC, neutrophil, and platelet counts are shown in Figure 2. And that of bilirubin are shown in Figure 3. At 1-month follow-up after discharge, the patient told us that she felt normal and did not have any discomfort. Written permission to publish this case report was obtained from the patient.

## 3 Discussion

Firstly, to establish the causal relationship between the most suspected medication (FA) and jaundice, neutropenia and aggravated thrombocytopenia, WHO Uppsala Monitoring Centre criteria^[2]^ was applied, the result showed that this adverse reaction had “probable” relationship with FA administration. Then, severity of this adverse reaction was assessed according to the Common Terminology Criteria for Adverse Events^[3]^ in grades 1 to 5 (mild to death), and the grade of our patient was 4.

The hematologic adverse effects caused by FA are rare. So far, there are 13 published cases of FA-induced cytopenia.^[4–10]^ These include 9 isolated neutropenia,^[4–7]^ 1 isolated thrombocytopenia,^[8]^ 2 neutropenia plus thrombocytopenia,^[9,10]^ and 1 leukopenia plus thrombocytopenia.^[10]^ In all those cases, cytopenia developed from 4 to 49 days after starting FA therapy, and resolved within 2 to 9 days after discontinuation of FA. In our report, the patient had a thrombocytopenia with platelet count of 66,000/μL prior to FA therapy, but neutropenia and aggravated thrombocytopenia developed 12 days after FA treatment, improvement of hematology index occurred 5 days after cessation of FA. Our patient was similar to what has been reported in the literature. El Kassar et al^[8]^ reported that in their patient's serum, an IgG antibody specifically recognizing platelet glycoprotein IIb/IIIa only in the presence of FA was identified which suggests that thrombocytopenia caused by FA was drug-induced immune thrombocytopenia. Similarly, Liao et al^[10]^ documented a case of thrombocytopenia, transfusion of platelet concentrates failed to improve it, and peripheral destruction was indicated by bone marrow examination showing megakaryocyte and myeloid hyperplasia. Bone marrow aspiration of our patient was performed, showing myeloid and megakaryocyte hyperplasia. And thrombocytopenia did not improve immediately after receiving transfusion of platelet concentrates, suggesting peripheral destruction. Therefore, given that bone marrow examination, the prompt recovery of hematology index of the patient after cessation of FA and previously published cases, it was speculated that FA-induced thrombocytopenia of our patient was immune mediated.

Apart from hematologic abnormalities, the patient also had abnormal liver function after FA use. In 1972, Copperman^[11]^ reported the 1st case documenting deterioration of hepatic function during therapy in a patient treated with IV FA. And jaundice was reported by Craig in 1974^[12]^ in a patient with pustular psoriasis who was treated with FA in combination with clindamycin. There are 44 cases of jaundice associated with FA between 1963 and 1980 to the Committee on Safety of Medicines.^[13,14]^ Subsequently, 10 cases of FA-associated jaundice and hyperbilirubinemia were also reported.^[15–19]^ FA is eliminated primarily by biliary excretion of various conjugative and cytochrome P450 oxidative metabolites.^[20]^ Wynn^[21]^ postulated that FA may interfere with pathways involved in the transport and secretion of bile pigment. Bode et al^[22]^ commented that there were at least 2 different machanisms involved in impairment of transport processes and hepatobiliary elimination by FA, direct inhibition of transport of multidrug resistance protein 2 (Mrp2) and bile salt export pump substrates and impairment by a decreased level of hepatic Mrp2. In addition, prolonged treatment with FA can result in a marked decrease of hepatic Mrp2 levels. In the case of jaundice corresponding with FA therapy,^[18]^ liver biopsy showed changes more consistent with cholestasis on histopathology and electron microscopy. The mechanism of jaundice in our patient is unclear due to the lack of liver biopsy and Mrp2 detection.

## 4 Conclusion

To our best knowledge, this is the 1st report of hematologic toxicities accompanying with hepatotoxicity induced by FA. Though the risk is rather low, it should not be overlooked. We recommend regular monitoring of full blood count and liver function when receiving FA therapy to avoid severe adverse effects. And take caution when using drugs excreted by the liver in patients with hepatitis B cirrhosis.

## Author contributions

**Conceptualization:** Zhong-Fang He.

**Data curation:** Lin Chen, Jian-Ping Zhang.

**Formal analysis:** Zhong-Fang He, Lin Chen, Jian-Ping Zhang.

**Funding acquisition:** Zhong-Fang He.

**Investigation:** Zhong-Fang He.

**Methodology:** Zhong-Fang He.

**Project administration:** Zhong-Fang He.

**Resources:** Zhong-Fang He.

**Supervision:** Zhong-Fang He.

**Writing – original draft:** Zhong-Fang He, Lin Chen, Jian-Ping Zhang, Qing-qing Wang.

**Writing – review & editing:** Zhong-Fang He, Lin Chen, Jian-Ping Zhang, Qing-qing Wang.
